# Supplementary material for: Comparing the Performance of Foundation Model Derived Embeddings with Traditional Approaches for Distant Metastasis Prediction in Head and Neck Cancer
Source: ArXiv. 2026 Jul 28:arXiv:2607.26276v1. Preprint. [Version 1] (PMC13484420)
Supplement: Supplementary file 1 [file NIHPP2607.26276v1-supplement-1.pdf]

VII. Supplemental Materials

Image Parameter	Radiomics	Model<br>Foundation	ViT
Image Format	Nifti	Nifti	Nifti
Resampling	None	None	1x1x1 mm
Cropping	None	Axial	ROI-based
Renormalization	None	None	Min-Max
Input Image	#x512x512 + GTVp	#x512x512	80x80x80 + GTVp
Features to classifier	1316	1408	256

Table S.1: Summary of input differences between the radiomics, foundation and ViT features.

Selected Radiomics Features
exponential_gldm_DependenceEntropy
exponential_gldm_DependenceNonUniformityNormalized
exponential_glrlm_GrayLevelNonUniformity
exponential_glrlm_LongRunEmphasis
exponential_glrlm_LongRunLowGrayLevelEmphasis
gradient_glrlm_GrayLevelNonUniformity
gradient_ngtdm_Busyness
original_glrlm_GrayLevelNonUniformity
original_shape_MajorAxisLength
original_shape_Maximum2DDiameterColumn
original_shape_Maximum2DDiameterRow
original_shape_Maximum3DDiameter
original_shape_SurfaceArea
square_gldm_DependenceNonUniformityNormalized
square_gldm_GrayLevelNonUniformity
square_glrlm_GrayLevelNonUniformity
square_glrlm_LongRunEmphasis
square_glrlm_LongRunLowGrayLevelEmphasis
square_glrlm_RunVariance
squareroot_glrlm_GrayLevelNonUniformity
wavelet-HHH_gldm_DependenceNonUniformityNormalized
wavelet-HLH_glrlm_GrayLevelNonUniformity
wavelet-HLL_gldm_GrayLevelNonUniformity
wavelet-HLL_glrlm_GrayLevelNonUniformity
wavelet-LHH_glcm_ClusterShade
wavelet-LHL_glcm_Correlation
wavelet-LLH_gldm_GrayLevelNonUniformity
wavelet-LLH_glrlm_GrayLevelNonUniformity
wavelet-LLL_glcm_SumSquares
wavelet-LLL_glrlm_GrayLevelNonUniformity

Table S.2: List of radiomics features selected by the mRMR algorithm for the SVM model.

Selected Foundation Embeddings
embedding_11
embedding_20
embedding_25
embedding_33
embedding_36
embedding_46
embedding_59
embedding_90
embedding_92
embedding_117
embedding_145
embedding_173
embedding_178
embedding_215
embedding_496
embedding_497
embedding_498
embedding_672
embedding_896
embedding_1096
embedding_1097
embedding_1098
embedding_1099
embedding_1100
embedding_1101
embedding_1102
embedding_1103
embedding_1184
embedding_1185
embedding_1272

Table S.3: List of foundation embeddings selected by the mRMR algorithm for the SVM model. The numbers correspond to the index position of the embeddings as they are output from the CT Foundation API.

Parameter	Grid Search Range
mRMR N features	[3,5,10,15,20,30,50,100,500]
Smote k neighbors	[3, 5, 10, 20]
SVM C	[0.1, 1, 10]
SVM kernel	[linear, rbf, poly]
SVM N degrees	[2,3,4]
SVM gamma	[0.01, 0.1, 1, 10, scale]

Table S.4: The grid search parameters for the traditional machine learning models. Separate grid searches were done for the SVM parameters.

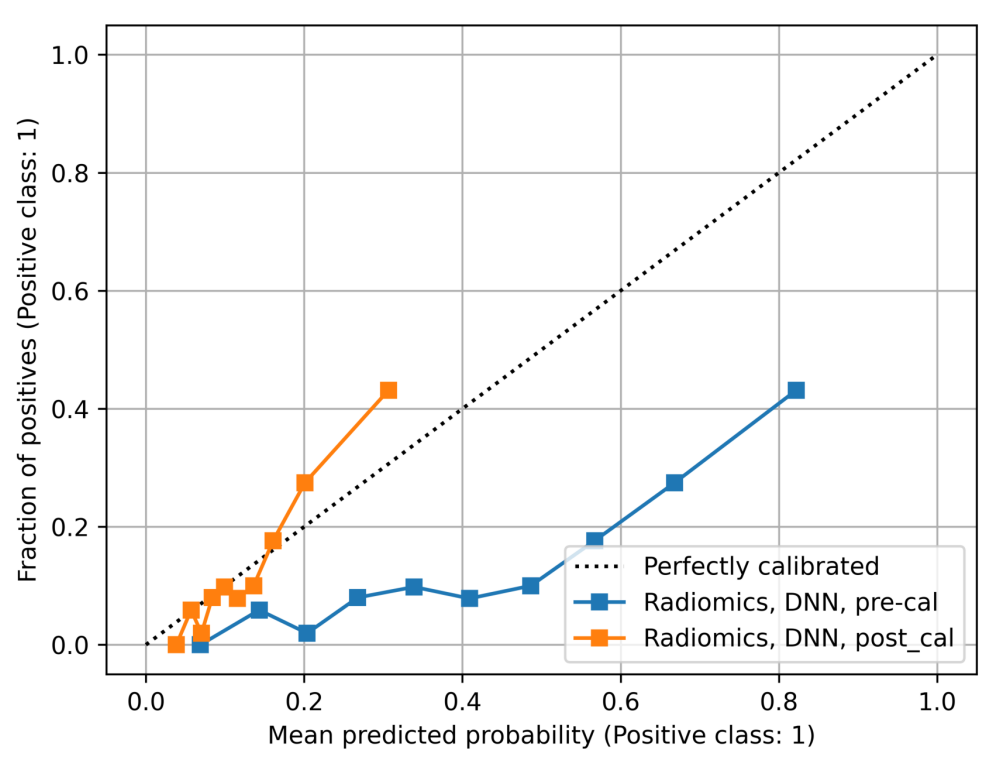

(a)

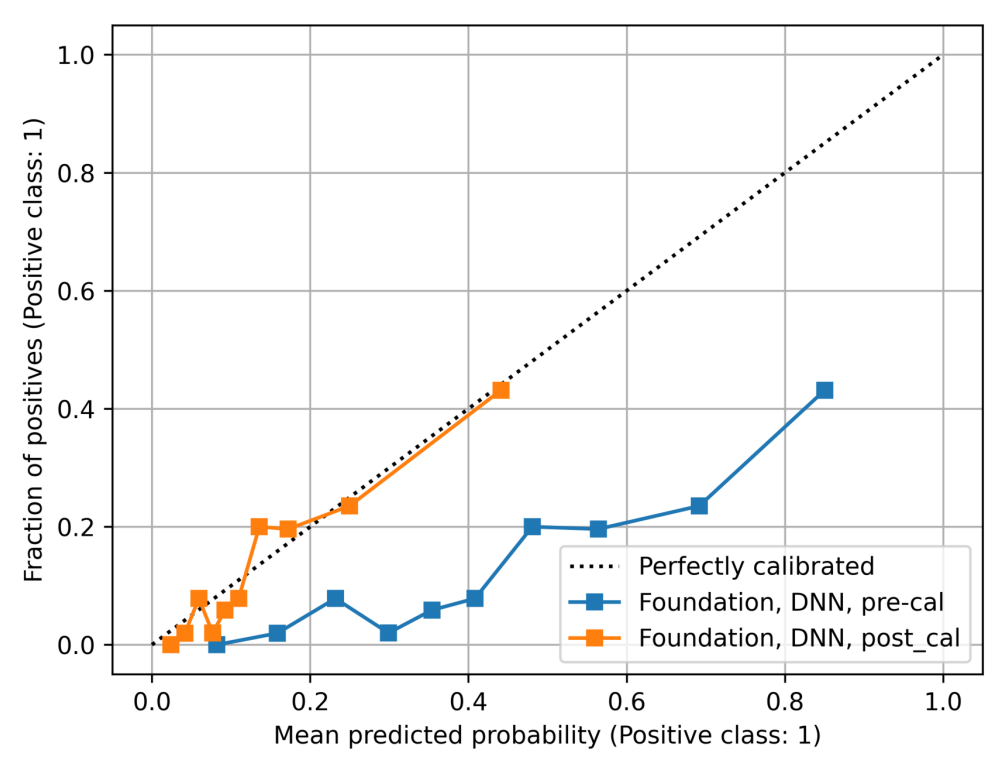

(b)

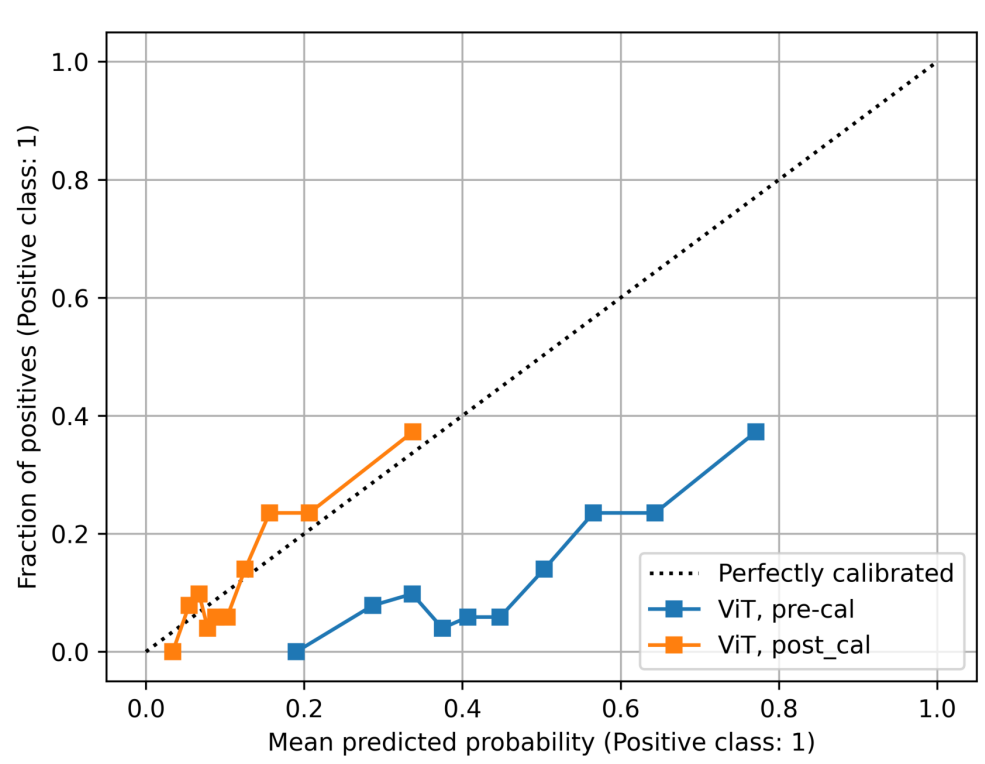

(c)

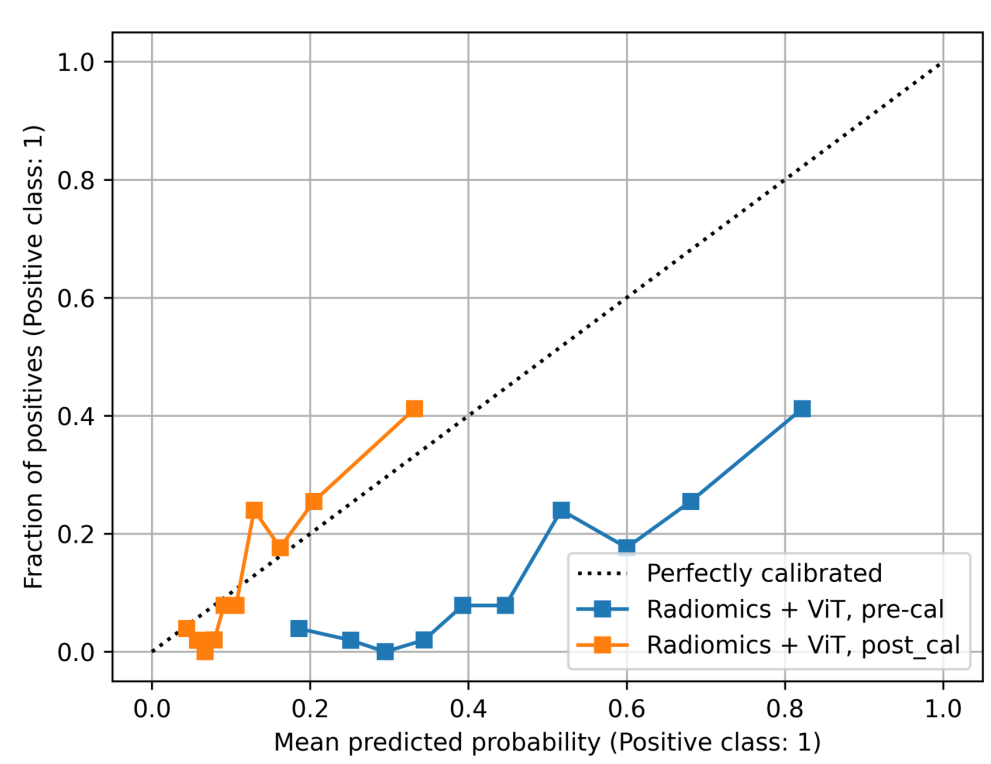

(d)

Figure S.1: Calibration curves for the DNN models before and after calibration.

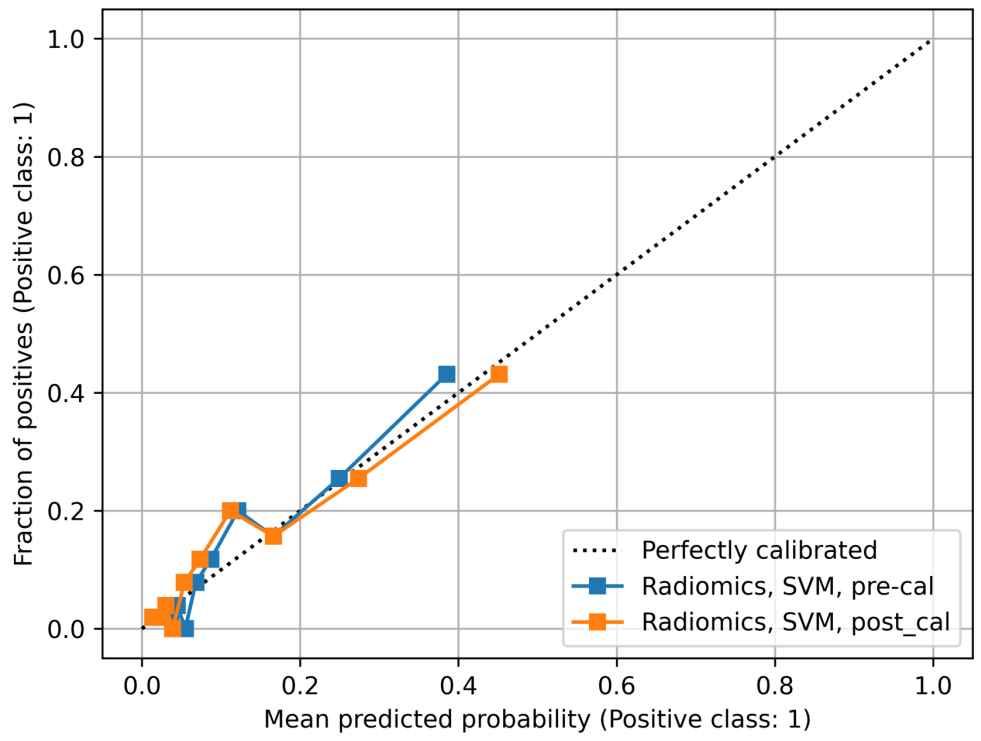

(a)

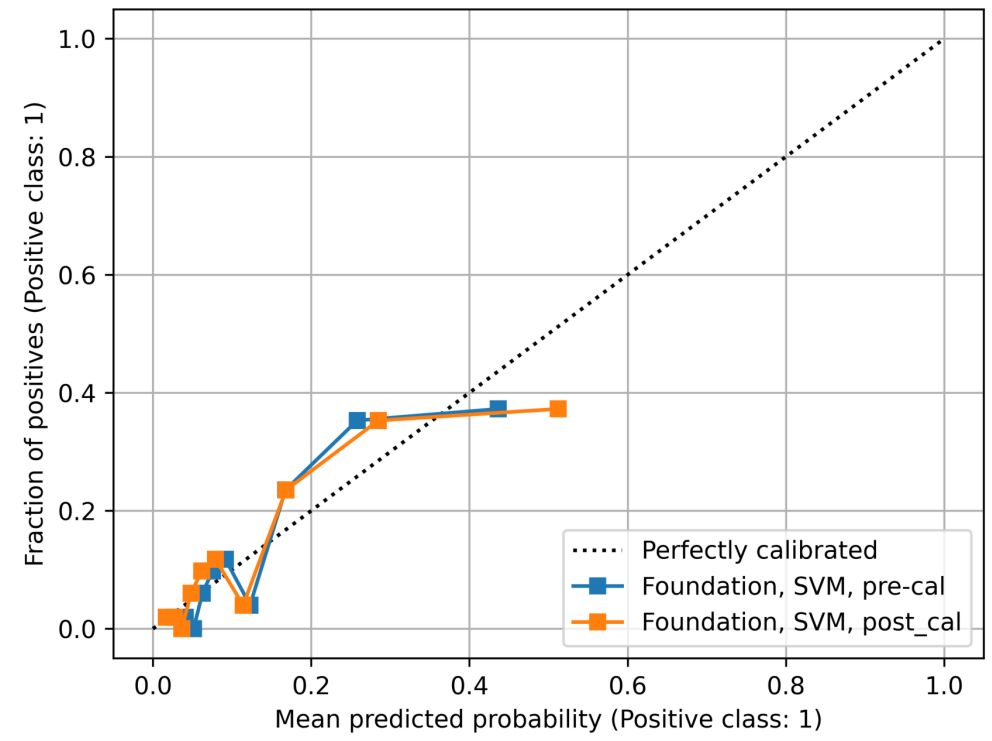

(b)

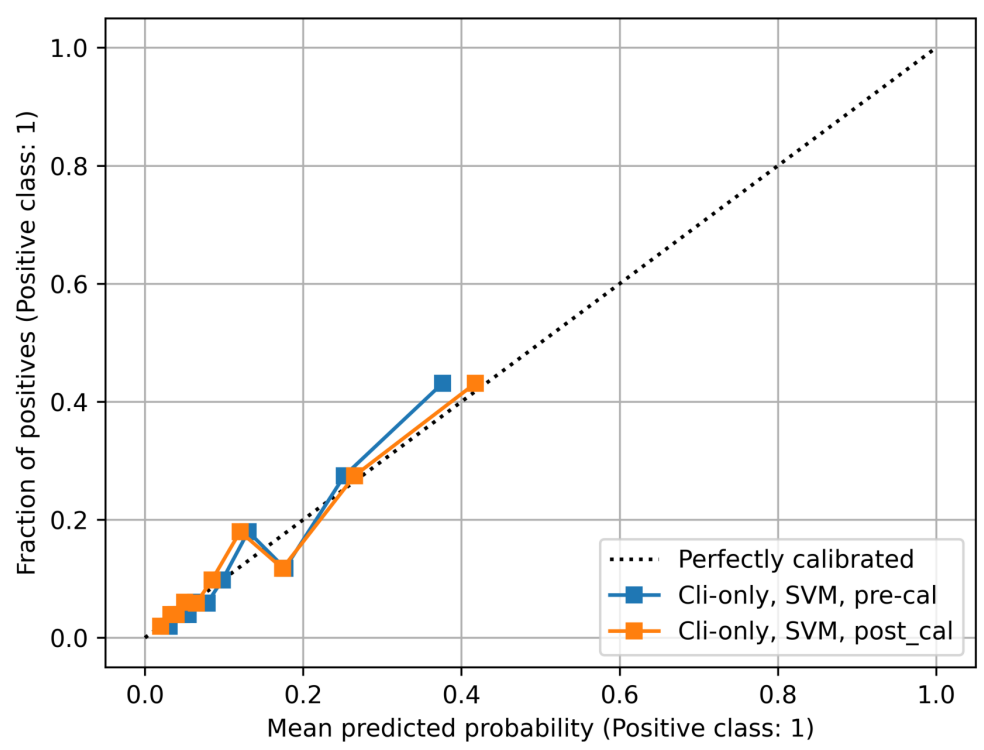

(c)

Figure S.2: Calibration curves for the SVM models before and after calibration.

Model	Threshold
Radiomics, DNN	0.139
Foundation, DNN	0.126
Image, DNN	0.128
Image+Radiomics, DNN	0.123
Radiomics, SVM	0.060
Foundation, SVM	0.064
No cli, SVM	0.073

Table S.5: Retuned thresholds for the various models after performing a calibration.

Model	CV Fold	AUC [95% CI]	AP	SEN	SPE
Foundation	0	0.784 [0.727, 0.841]	0.426	0.657	0.755
	1	0.776 [0.718, 0.834]	0.417	0.657	0.757
	2	0.789 [0.733, 0.844]	0.412	0.687	0.717
	3	0.789 [0.732, 0.845]	0.421	0.687	0.753
	4	0.78 [0.724, 0.837]	0.379	0.806	0.669
	Avg ±std	0.784 ± 0.006	0.411 ± 0.019	0.699 ± 0.062	0.730 ±0.038
Radiomics	0	0.78 [0.723, 0.837]	0.343	0.836	0.558
	1	0.754 [0.692, 0.816]	0.357	0.552	0.791
	2	0.779 [0.721, 0.838]	0.379	0.821	0.540
	3	0.763 [0.703, 0.823]	0.387	0.627	0.760
	4	0.739 [0.672, 0.806]	0.359	0.582	0.741
	Avg ±std	0.763 ± 0.017	0.365 ± 0.019	0.684 ± 0.135	0.678 ± 0.119
ViT	0	0.741 [0.676, 0.806]	0.344	0.657	0.723
	1	0.727 [0.661, 0.792]	0.332	0.672	0.683
	2	0.756 [0.693, 0.819]	0.382	0.701	0.735
	3	0.757 [0.693, 0.82]	0.358	0.731	0.644
	4	0.757 [0.693, 0.822]	0.366	0.716	0.707
	Avg ±std	0.748 ± 0.013	0.356 ± 0.019	0.695 ± 0.030	0.698 ± 0.036
Radiomics + ViT	0	0.781 [0.722, 0.841]	0.367	0.701	0.739
	1	0.8 [0.747, 0.853]	0.398	0.896	0.562
	2	0.799 [0.747, 0.85]	0.370	0.896	0.583
	3	0.758 [0.694, 0.821]	0.374	0.657	0.728
	4	0.777 [0.719, 0.835]	0.375	0.731	0.687
	Avg ±std	0.783 ± 0.017	0.377 ± 0.012	0.776 ± 0.112	0.660 ± 0.082

Table S.6: Performance of the testing set on the selected DNN models from each cross-validation fold, before applying calibration. The AUC, average precision, sensitivity, and specificity are provided

Model + Selection	AUC [95%CI]	AP	SEN	SPE
Foundation Models				
Foundation + Contrast	0.780	0.372	0.750	0.685
Foundation + No Contrast	0.847	0.667	0.909	0.682
Foundation + Male	0.790	0.437	0.780	0.668
Foundation + Female	0.786	0.238	0.750	0.763
Foundation + No clinical	0.707	0.350	0.687	0.562
Foundation	0.791	0.412	0.776	0.685
Radiomics Models				
Radiomics + Contrast	0.762	0.334	0.679	0.712
Radiomics + No Contrast	0.822	0.617	0.727	0.750
Radiomics + Male	0.770	0.392	0.678	0.704
Radiomics + Female	0.766	0.318	0.750	0.750
Radiomics + No clinical	0.718	0.261	0.642	0.644
Radiomics	0.772	0.379	0.687	0.712

Table S.7: Performance of two DNN models using radiomics and foundation embeddings as input for different selections of patient populations and for separately trained models that remove the use of clinical features. The original model performances are provided for comparison.
